# ID 256 - Estudo de Custos das Mulheres que Fazem o Exame Citopatológico nas Unidades Básicas de Saúde (UBS) do Distrito Federal – Brasília

**DOI:** 10.5327/2237-9622.2025.v34s1.256

**Published:** 2025-11-25

**Authors:** Aline Leal Gonçalves Creder Lopes, Raquel Santos de Souza, Carmen Nila Phang Romero Casas, Tainá Raiol Alencar

**Keywords:** custos, detecção precoce, rastreamento, Papanicolau, câncer de colo de útero

## Abstract

**Introdução:**

O câncer do colo do útero (CC) é uma das principais causas de morbidade e mortalidade entre as mulheres, tanto no Brasil quanto globalmente. Embora o País tenha alcançado uma cobertura de exames citopatológicos (teste de Papanicolau) de cerca de 80%, é essencial melhorar a eficácia do rastreamento para reduzir a mortalidade relacionada à doença. O Brasil está atualmente reformulando seu programa de rastreamento nacional, adotando estratégias organizadas e incorporando testes moleculares de HPV. Para implementar essas novas abordagens com sucesso, é fundamental conhecer o impacto econômico que representa para as mulheres a realização do exame citopatológico convencional. Isso permitirá o desenvolvimento de estratégias eficazes para promover a adesão ao programa de rastreamento. Ao mesmo tempo, poderá subsidiar os gestores com informações econômicas adequadas que justifiquem o investimento de novos recursos, visando a melhores resultados em saúde.

**Método:**

Foi realizada uma pesquisa qualitativa exploratória por meio de entrevistas com mulheres que realizaram o exame citológico em Unidades Básicas de Saúde (UBS) no Distrito Federal. Os critérios de inclusão foram maiores de 18 anos, presentes nas unidades durante as entrevistas e aceitando participar. O critério de saturação foi utilizado para finalizar a coleta de dados. Foi elaborado um roteiro com questões sobre tempos, movimentos e recursos utilizados pelas mulheres para se deslocar até a UBS, seguindo o fluxo dos processos para realização do exame citológico. O questionário com 28 perguntas, originalmente em inglês, teve adaptação de idioma para o português e foi aplicado por estudantes de medicina, previamente treinados. As participantes foram anonimizadas e identificadas por letras e números. Uma análise estatística descritiva foi utilizada para a síntese de resultados.

**Resultados:**

Foram realizadas 31 entrevistas com mulheres com mediana de idade de 41 anos, e 74% possuíam ensino médio ou superior. Em sua maioria, as participantes eram casadas ou divorciadas (84%) e tinham renda familiar de até R$ 1.412,00. Quase todas as participantes (93,5%) relataram ter realizado a citologia previamente, das quais 25,8% fazem o exame anualmente, e 48,4% tiveram de uma a duas gestações. Com relação ao tempo de deslocamento até a UBS, foi em média de 18 min (IQR: 10-30 min); o tempo de agendamento apresentou média de 21 min (IQR: 10-30 min); o tempo de espera para realização do exame, uma média de 51 min (IQR: 20-60 min); e o tempo de consulta, considerando a realização do exame citopatológico, uma média de 31 min (IQR: 20-40 min). A maioria das mulheres (67,7%) compareceu à UBS sem acompanhante, enquanto uma menor parcela (32,3%) estava acompanhada de outro adulto.

**Conclusão:**

Atualmente o modelo de rastreamento de câncer de colo de útero por meio do exame citopatológico é oportunístico e, portanto, não organizado. O tempo dispendido pelas mulheres desde o deslocamento à unidade de saúde até a realização do exame representa um custo de oportunidade perdido para a realização de qualquer outra atividade produtiva, o que impacta o ingresso familiar. Isso pode explicar, em parte, a baixa adesão ao exame preventivo. A reformulação do programa de rastreamento para CC com a incorporação de testes moleculares e a transição para o rastreamento organizado poderá estimar o custo-efetividade das novas estratégias em comparação com a citopatologia convencional. Espera-se que o estudo contribua para essas avaliações econômicas.

